# Accessible Prosthetic Arms: Victoria Hand Project and The Impact of 3D Printing

**DOI:** 10.33137/cpoj.v6i2.42142

**Published:** 2023-12-22

**Authors:** N Dechev, K Knights, K Arklie, M Martindale, M Peirone

**Affiliations:** 1 Biomedical Designs and Systems Laboratory, University of Victoria, Victoria, V8P 5C2, Canada.; 2 Department of Mechanical Engineering, University of Victoria, Victoria, V8P 5C2, Canada.; 3 Victoria Hand Project, 3060 Westridge Place, Victoria, BC, V9E 1C8, Canada.

**Keywords:** 3D Printing, 3D Printed Prosthetic Arm, 3D Printed Hand, Additive Manufacturing, Charity, Humanitarian

## Abstract

Victoria Hand Project (VHP) is a Canadian charity with a mission to provide 3D printed prosthetic arms to people in-need across the world, by partnering with prosthetic care providers. This article explores the journey of VHP, sharing insights, lessons learned, ongoing directions, and the impact of 3D printing on prosthetic care for people with upper-limb amputation. Benefits such as affordability and customization are explored, as well as the challenges encountered, including quality control and the steep learning curve associated with working in the digital 3D space. Through this article, the potential of 3D printing to continue to transform the field of assistive technology and prosthetic and orthotic applications is underscored, especially when used for collaborative, humanitarian initiatives.

## INTRODUCTION

The field of prosthetics and orthotics has witnessed transformative changes in recent years, driven by advances in rapid prototyping technologies (i.e., 3D printing, 3D scanning, and 3D computer aided design (CAD)). This article describes the experience of Victoria Hand Project (VHP) using these technologies to implement low-cost prosthetic applications, including the benefits and challenges encountered. VHP's journey has leveraged this new technology, in conjunction with tried-and-true traditional manufacturing methods, to provide prostheses to people with upper limb amputations, who face with limited access to prosthetic care.

## VICTORIA HAND PROJECT: A BRIEF OVERVIEW

VHP is a registered Canadian charity with a mission to help people in-need receive prosthetic arms. Incorporated in 2015, VHP partners with prosthetic care professionals worldwide. Partners are provided with equipment, training, and on-going support to enable them to create and provide prosthetic arms in their own clinics, for people in their own community. This model lays the groundwork for local, sustainable, and on-going care for people with upper limb amputations who may not be able to receive a prosthetic device otherwise. Receiving a Victoria Hand is transformative: functional prosthetic arms are a vital tool to help people regain independence, hope, and opportunities to live more fulfilling and happier lives. The impact goes far beyond the recipient, with positive effects for their families, caretakers, and for increased clinic capacity.

The VHP 3D printed prosthetic system has evolved over the years, with many thousands of hours devoted to engineering design and testing. There has been on-going close consultation with Canadian, US, and international prosthetists, as well as feedback from hand recipients, which has informed the design direction. VHP has developed a range of prosthetic arm systems, and continually worked to improve the fit, cosmetic appearance, and function of these. Each prosthetic system is customized by selecting from dozens of possible components such as various: terminal devices, wrists, custom limb sockets, and harness components. These are selected, combined, and further customized to suit the unique needs of each person. Terminal devices options include: a voluntary close hand, a voluntary open hand, a cosmetic-passive hand, and a voluntary close pediatric hand. Recipients are encouraged to pick and choose between features that work best for them and their lifestyle. VHP offers 3D printed limb sockets for various amputation levels, including trans-radial options for below-elbow amputations, using a PLA (polylactic acid) socket for longer residual limbs, and a flexible Polypropylene inner socket with a rigid PLA outer socket for shorter residual limbs. Additionally, a trans-humeral system is available for people with above-elbow amputations, comprising a PLA upper limb socket, stainless steel elbow mechanism, and PLA forearm. Given all these possible options and combinations, VHP has recently developed software to aid prosthetists in the workflow, and also integrated 3D CAD to facilitate socket creation. In this way, by partnering with local clinics, and training them in this production system, VHP's approach allows for high-quality care and builds in-country institutional capacity. To date, VHP has provided over 300 Victoria Hand prosthetic arms worldwide, trained over 50 clinicians in rapid prototyping technology, and established 11 on-going partnerships in: Cambodia, Canada, Egypt, Guatemala, Haiti, Kenya, Nepal, Pakistan, Uganda, Ukraine, and the United States. Personal anecdotes from recipients demonstrate the benefits from increased function, increased self-esteem in public, to becoming more independent at home and work.

It is important to explain that VHP systems were specifically designed to minimize cost and thereby maximize accessibility worldwide, where they are approximately $100–150 USD in materials depending on the system. Designing prosthetic devices to meet such a low cost has major implications and has driven the nature of the tools and methods used, as explained in this paper.

## DESIGN PHILOSOPHY & BENEFITS OF 3D PRINTING

### Customizable Socket Creation:

The workflow of creating a 3D printed socket begins with the prosthetist taking anatomical measurements of both upper-limbs, making a traditional plaster limb impression of the residual limb, creating a positive cast, rectifying it, and then 3D scanning the positive impression. This retains the clinicians' sense of tactile feedback, for them to incorporate space or padding in the socket (via positive impression) as needed for each patient. After scanning, the 3D scan is then imported into VHP's software, which guides the clinician through the process of turning the impression into a 3D printable limb socket. Employing 3D printing introduces benefits: This frees Prosthetist and Technician time, to concentrate their expertise on patient care. The 3D printers can fabricate parts without clinician intervention, where a large limb socket may print in 8–10 hours overnight without any interaction needed. If terminal devices are printed and assembled ahead of time and kept “in stock”, a patient could visit a clinic, be measured and cast, and receive a custom prosthetic system the next day. This process reduces equipment needs, since there is no longer a need for oven heaters, draping hot plastic sheets, vacuum systems, trimming and grinding equipment, or other infrastructure for making traditional sockets. It allows for complex shapes to be manufactured directly into sockets (connection points, cable guides, wrist connectors) thereby digitally integrating several traditional components/features into a single socket. This reduces traditional inventory since components/features can be printed on-demand. The socket interior shape is replica of the impression, whereas the exterior body is a parametric shape by using patient measurements, where the average thickness is 8mm.

### Rapid Prototyping for Quick Production:

VHP's prosthetic devices are made by using a variety of different rapid prototyping technologies and widely available parts. The primary design goal is low-cost, highly functional, durable prosthetic arm systems that can be built on-site in various countries worldwide. This is achieved by using 3D printing, 3D scanning, 3D CAD, and also 2D laser-cutting (a more recent rapid prototyping technology). Several laser-cut stainless-steel components (cut from 1.9 mm stainless steel sheet) are used in the terminal devices (hands), elbow, back-lock, and for small, high-stress parts within the prosthesis, to ensure durability and long life. 3D printing allows for manufacturing of parts in small batches, without the need to inventory hundreds of different parts (as is traditionally done). Printing on-demand parts mean less supply-chain disruption, fewer shipping and import fees, fewer delays, and enables on-site service/repair in hard-to-reach places in the world.

### Cost Effective Design:

Rapid prototyping allows for low-cost production of prosthetic devices. Materials for a complete VHP prosthetic system, cost approximately $100 to $150 USD, compared to conventional devices that cost several thousands.^1,2^ VHP provides stipends (per fitting) to the international partner clinicians and technicians, who are each paid $100 to $150 USD to support their business and livelihood, bringing the total cost for provision of a VHP system to between $300-$450 USD depending on the location and configuration. This is approximately 10% of the estimated cost to receive a body-powered hook.^2^ VHP's partnership agreement offers the devices to prospective amputees on a pay-what-you-can model, and often free to those most in need. Since VHP is a charity, development costs do not need to be recovered, further keeping device costs low.

### Rapid Global Design Updates:

With rapid prototyping, design improvements and feature updates to prosthetic devices can be transmitted digitally and instantly. Since parts are 3D printed on-demand, design improvements can be implemented rapidly. When international clinical partners share patient feedback that warrants a design change, VHP engineers can make the change in Canada, build it, test it and then push the new designs (as digital files) via the software to partners. Version control is an important aspect for VHP, where each 3D printed part has a date-based model number inscribed.

### In-Country Production:

On-site, on-demand 3D printing also enables the rapid replacement of worn/broken parts, reducing downtime for individuals and clinics. The aforenoted version control also preserves ability to re-print legacy (older) 3D parts for repair of older systems. Technicians and clinicians can quickly make adjustments and repairs. This ensures that people travelling to the clinic can receive care and maintenance promptly, minimizing the challenges of travel and long wait times without their device.

## DETAILS AND CHALLENGES OF 3D PRINTING

VHP collaborates with the University of Victoria's Engineering Faculty (Biomedical Design and Systems Laboratory) to perform extensive mechanical and other testing on 3D printed parts before deployment. Through this process a wealth of information has been learned over the past decade, where some recommendations are summarized here:

### Design for 3D Printing:

The most significant challenge in 3D printing for prosthetics or orthotics applications is ensuring the strength and durability of the devices compared to traditionally manufactured ones. VHP prosthetics are made using the FDM (Fused Deposition Modelling) 3D printing method, which is the successive addition of thin layers (0.4 mm to 0.6 mm) of molten plastic material, to build up a desired part layer-by-layer. As such, the tensile strength of 3D printed parts is different along different directions (non-isotropic), where there is relatively high-strength in-plane of the layer, and relatively low-strength between the layer planes. This is referred to as inter-layer adhesion. Different materials, and variability in print settings (layer height, nozzle diameter and temp, infill density, part-orientation, and print speed) will also impact the final part's strength. Given the limitation of inter-layer adhesion, VHP makes extensive use of small threaded bolts and nuts, within various prosthetic parts. This serves to maintain compression between layers, to maximise tensile and bending strength. This is used within the various terminal devices, the wrists, and the elbow. VHP also makes extensive use of metal components for very small, high-stress parts. For example, 2 mm diameter pins of various lengths for rotational elements in the fingers, within the palm, the wrist, the force doubler and other components. Recently, the incorporation of laser-cut stainless-steel components has been introduced to function as internal structural elements, akin to the bones (phalanges) within the fingers, as well as structural elements within the hands, the elbow and the force doubler. This combination of 3D printed PLA and metal parts, provides the distinct advantages of each approach, resulting in devices that exhibit optimal functionality, aesthetics, cost-efficiency, and durability.

### Materials:

PLA (polylactic acid) material is used for rigid/hard components of the prosthesis, including sockets. Although many dozens of materials have been extensively tested, it was found that PLA is by far the best material for various factors: Biocompatible (body contact wearable safe) for some brands,^3^ most reliable material (least 3D print failures, least jammed nozzles, etc), most durable material (3-5+ years of service life), lowest cost and most widely available, great strength (almost as strong as ABS) vs cost, best 3D printed results over a range of print settings. VHP has great success with two different PLA materials: BASF Forward AM PLA material which is certified body-safe,^3^ and FormFutura EasyFil PLA, where the black color material has the highest strength (rigorously tested by VHP).

### Testing:

Rigorous benchtop testing of the various devices has been done, using several or more replicate tests. Some examples include: Finger testing, where a finger (within the prosthesis) is loaded at the tip in extension, where on average a single finger can hold 80 lbs (356 N) of weight without failure. Wrist testing, where the ball and socket wrist is loaded such that the prosthesis can hold 43 lbs (190 N) at a distance of 7.5 cm from wrist (Torque of 14 N-m), before failure. Note wrist slip rotation will occur at 11 lbs (47 N, Torque of 4.9 N-m). Elbow joint testing, where the prosthesis is loaded at the distal end of the forearm (19 cm from elbow), with an average weight of 18 lbs (80 N) before slip rotation (Torque of 15.2 N-m). The voluntary close hand (VC300) weighs 1.5 lbs (0.68 kg), with the total transradial prosthetic system, including the hand, wrist, transradial socket, cabling and harness, weighing approximately 3 lbs (1.4 kg). Similarly, the transhumeral system consisting of a VC300, the wrist, a forearm, elbow mechanism, upper transhumeral socket, cabling and harness is a total of approximately 5 lbs (2.3 kg).

### 3D Printers:

There is a large variability amongst 3D printers, with hundreds of different models available. VHP has found the best value in terms of performance-to-cost with the Ultimaker brand of 3D printers, in particular the Ultimaker 2+ Extended and the Ultimaker Connect, as well as the Prusa XL 3D printers. These machines exhibit great reliability in various environments (dust, temperature, humidty), handle thousands of print hours, are easy serviceability with low cost, and have good build-volume and build height (fundamentally important for creating large/long sockets). By exclusively utilizing these 3D printers, predetermined print settings, and the aforementioned PLA material, VHP ensures consistent and well-controlled 3D printed parts. This meticulous standardization not only enhances production efficiency but also guarantees the reliability and quality of each prosthetic arm, as mandated by partnership agreements with international partners.

### Challenges:

A major challenge faced by VHP was training partner clinicians in 3D digital technology (printing, scanning, CAD), and integrating that with conventional prosthetic fabrication methods. There is a steep learning curve associated with Computer Aided Design (CAD), working with and visualizing 3D meshes on-screen, and 3D scanning. Many clinicians are already busy with their day-to-day work and may not have the time available to learn these new skills in-depth. VHP recognises the importance of making this technology accessible and easy-to-understand for clinicians and has created comprehensive training procedures and as well as in-house software programs dedicated to making the learning process easier and more intuitive. By providing clinicians with the necessary skills and knowledge to work with 3D-printing technology, VHP provides them with new tools to use in conjunction with traditional methods.

## CALL TO ACTION

Readers (prosthetists, technicians, and clinicians) are encouraged to consider the techniques and methods introduced in this paper. Rapid prototyping can contribute in significant or subtle ways to the construction of prostheses, and various possible approaches exist. Consequently it can enhance lives and contribute to the advancement of prosthetic technology. The authors can share more specific details with interested parties upon request.

## DECLARATION OF CONFLICTING INTERESTS

**Dr. Nick Dechev** is the founder of Victoria Hand Project, a charitable organization dedicated to providing affordable prosthetic care to underserved populations. **Kelly Knights** is the Chief Operating Officer, **Kim Arklie** is Mechanical Engineer, **Michelle Martindale** is Biomedical Systems Designer, and **Michael Peirone** is Chief Executive Officer at the Victoria Hand Project.

## AUTHOR CONTRIBUTION

**Nick Dechev** emphasizing design philosophy and the benefits of 3D printing,

**Kelly Knights,** and **Kim Arklie** focusing on technical aspects, **Michelle Martindale**, focusing on rapid global design updates, and **Michael Peirone** focusing on details and challenges of 3D printing.

All authors provided final approval for the version to be published and agreed to be accountable for all aspects of the work. The experiments were planned and carried out collaboratively by **N.D.**, **K.K., K.A., M.M.**, and **M.P**. including data acquisition, analysis, and interpretation. All authors, including **N.D., K.K., K.A., M.M.**, and **M.P.**, actively contributed to the interpretation of results, sample preparation, and critical feedback, helping shape the research, analysis, and manuscript.

## SOURCES OF SUPPORT

This project has received support from Choose Love (2023), Fauji Foundation (2023), TD Bank (2019), Google.org (2017), Grand Challenges Canada (2014, 2016), and NSERC (2014).

## CORRESPONDING AUTHOR SCIENTIFIC BIOGRAPHY

**Figure:**
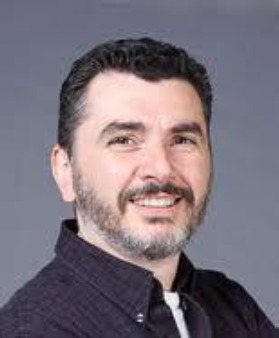


Nick Dechev is an Associate Professor in the Department of Mechanical Engineering at the University of Victoria, and former Program Director for Biomedical Engineering. He has a strong background in biomedical systems design, mechatronics, robotics and automation, assistive technology, and 3D printing. Nick's research focuses on developing innovative solutions to enhance the lives of individuals with disabilities, particularly in the field of prosthetic and orthotic devices. He is the founder of Victoria Hand Project, a charitable organization dedicated to providing affordable prosthetic care to underserved populations.
